# Perception of string quartet synchronization

**DOI:** 10.3389/fpsyg.2014.01115

**Published:** 2014-10-14

**Authors:** Alan M. Wing, Satoshi Endo, Tim Yates, Adrian Bradbury

**Affiliations:** ^1^SyMoN lab, School of Psychology, University of BirminghamBirmingham, UK; ^2^The Chair of Information-oriented Control, Department of Electrical Engineering and Information Technology, Technische Universität MünchenMünchen, Germany; ^3^Health and Safety LaboratoryBuxton, UK; ^4^Royal Academy of Music, University of LondonLondon, UK

**Keywords:** timing, synchronization, string quartet, feedback correction, listening test

## Abstract

Timing variation in small group musical performance results from intentional, expressive, and unintentional, error components in individual player timing. These timing fluctuations produce variability in between-player note asynchrony and require timing adjustments to keep the ensemble together. The size of the adjustments relative to the asynchrony (correction gain) affects the amount and nature of asynchrony variability. We present new listening tests to estimate thresholds for perception of between-player asynchrony variability and to determine whether listeners use differences in the nature of the variability, as well as in its magnitude, to judge asynchrony. In two experiments, computer-simulated ensemble performances of a 48-note excerpt from Haydn Op. 74 No. 1 were generated. Between-player note asynchrony was systematically manipulated in terms of level of within-player timing variability (Experiment 1) and correction gain (Experiment 2). On each trial, participants listened to two samples, one (“target”) with more between-player asynchrony variability than the other (“test”), and reported which was “less together.” In both experiments, the test sample correction gain was fixed at the statistically optimal value of 0.25 and the within-player timing variability was minimal (zero except for random variability in the initial note). In Experiment 1 the target correction gain was fixed at 0.25 and the timing variability was adjusted over trials by a staircase algorithm designed to converge on the level of asynchrony variability giving 75% correct identification. In Experiment 2 the timing variability in the target was set at half that in Experiment 1 and the correction gain was varied to converge on 75% correct identification. Our results show that the between-player asynchrony variability giving 75% correct identification in Experiment 2 was significantly lower than in Experiment 1. This finding indicates that people are sensitive to both the degree of variance and the micro-structure of the time-series of the asynchronies caused by differences in correction gain when judging lack of togetherness in quartet performance.

## INTRODUCTION

Timing in ensemble musical performance is variable, both within and between players. A large part of this variability is intentional, arising from scored differences in note duration or players’ intentional departures from the score as part of musical interpretation, e.g., lengthening of notes when slowing the tempo at the end of a piece. However, some unintentional variation in timing may also be expected in the duration of notes (e.g., the longer the note, the greater is likely to be the timing variability) or as a result of tempo fluctuations. Although players may seek to reduce the level of unintentional variation by practice, it cannot be eliminated completely. In consequence, there will be small asynchronies between the note onsets of the various players. These asynchronies will vary from note to note, which may affect the listener’s perception of ensemble and this is the focus of the present paper.

Recordings of various ensembles have shown that between-player asynchrony variability [measured in terms of SD] in professional ensembles playing various pieces from the repertoire [metronome indications between 40 and 130 beats per minute, (bpm)] is typically in the 10s of milliseconds range, reducing with increasing tempo (Rasch, 1979, 1998). In a string trio (violin, viola, and cello) the average between-player asynchrony SD was 49 ms (at an average 79 bpm) while the corresponding value for a wind ensemble (oboe, clarinet, bassoon) was 32 ms (at 88 bpm). The range was 24–73 ms. Recently Wing et al. (2014) reported figures for asynchrony SD of 24 and 28 ms (at 157 bpm) for two string quartets (first and second violin, viola, and cello). In the current study we develop a listening test to examine the perceptibility of differences in the variability of between-player asynchrony in music performance. We ask: what is the threshold for perceiving a difference between the asynchrony variability of two otherwise identical performances of a string quartet?

**FIGURE 1:**
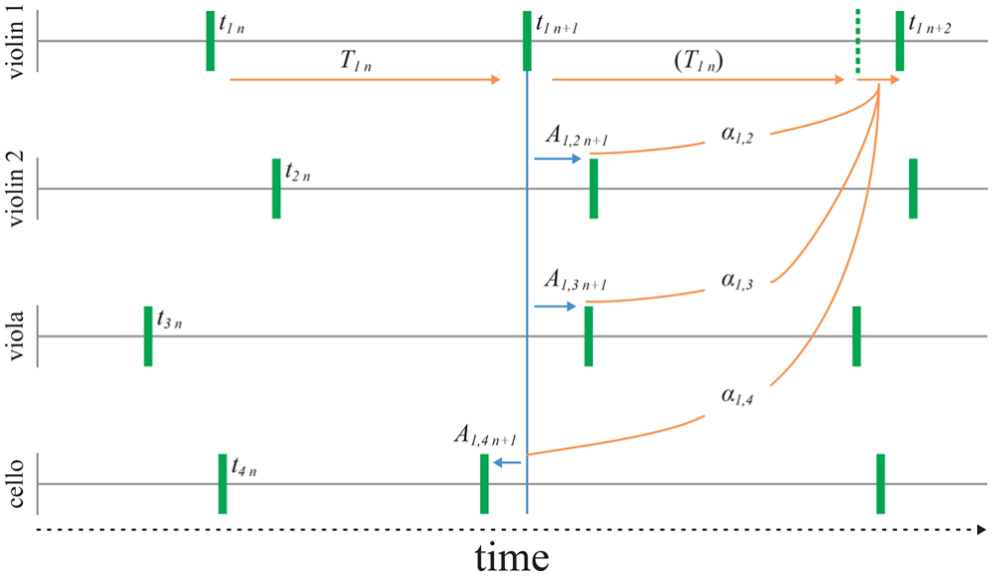
**Asynchrony feedback correction model of quartet synchronization.** The next event time, *Tn* is derived from the current asynchrony against each other player, *An* and the correction gain (α) shown for violin 1. Random timing variability ($\sigma_{\epsilon }^{2}$) is assumed to affect the intervals, *Tn*.

Although there is between-player asynchrony variability in quartet playing it appears to be stable over the course of a performance. It does not, for example, increase progressively throughout a piece of music (Moore and Chen, 2010). This implies that a control mechanism limits any tendency of ensemble players to drift apart when the individual players are prone to variability in their timing. Recently we proposed classical string quartets use first-order linear feedback correction to maintain ensemble (Wing et al., 2014). Over successive notes intended to be played together, each player uses the set of asynchronies with each of the other players at a given note to make a correction to the timing of the next note (see **Figure 1**). This correction is assumed to be a sum of proportional corrections:

(1) $$t_{i,n}=t_{i,n-1}+T_{i,n-1}-\overset{4}{\underset{j=1,j\neq i}{\Sigma }}\alpha_{ij}\left(t_{i,n-1}-t_{j,n-1}\right)+\epsilon_{i,n}\,\;i=1,...4\,$$

where t_i,n_ and t_i,n-1_ are current and previous observed note onset event times for Player *i*, T_i,n-1_ represents the timekeeper interval, α_ij_ refers to the correction gain applied by Player *i* for the asynchrony (*t*_*i*,*n*–1_ - *t*_*j*,*n*–1_) with Player *j*, and 𝜀_i,n_ is a random noise term identified with timing variability of intervals generated by an assumed internal timekeeper (Wing and Kristofferson, 1973).

If the sum of the proportional terms in Eq. (1) lies between 0 and 0.5, the asynchrony on the next note tends to reduce and the asynchrony time series is stable. The size of the correction used by one player may differ for each of the other players. Thus more weight might be given to one player than another, for example because the timing of one is perceived as being more reliable than the other. Alternatively, the weight assigned to one player may be greater because that part is perceived as having greater musical significance (e.g., the melodic line) and it is considered important for non-leading parts to prioritize timing corrections with that leading part over corrections with each other.

**FIGURE 2:**
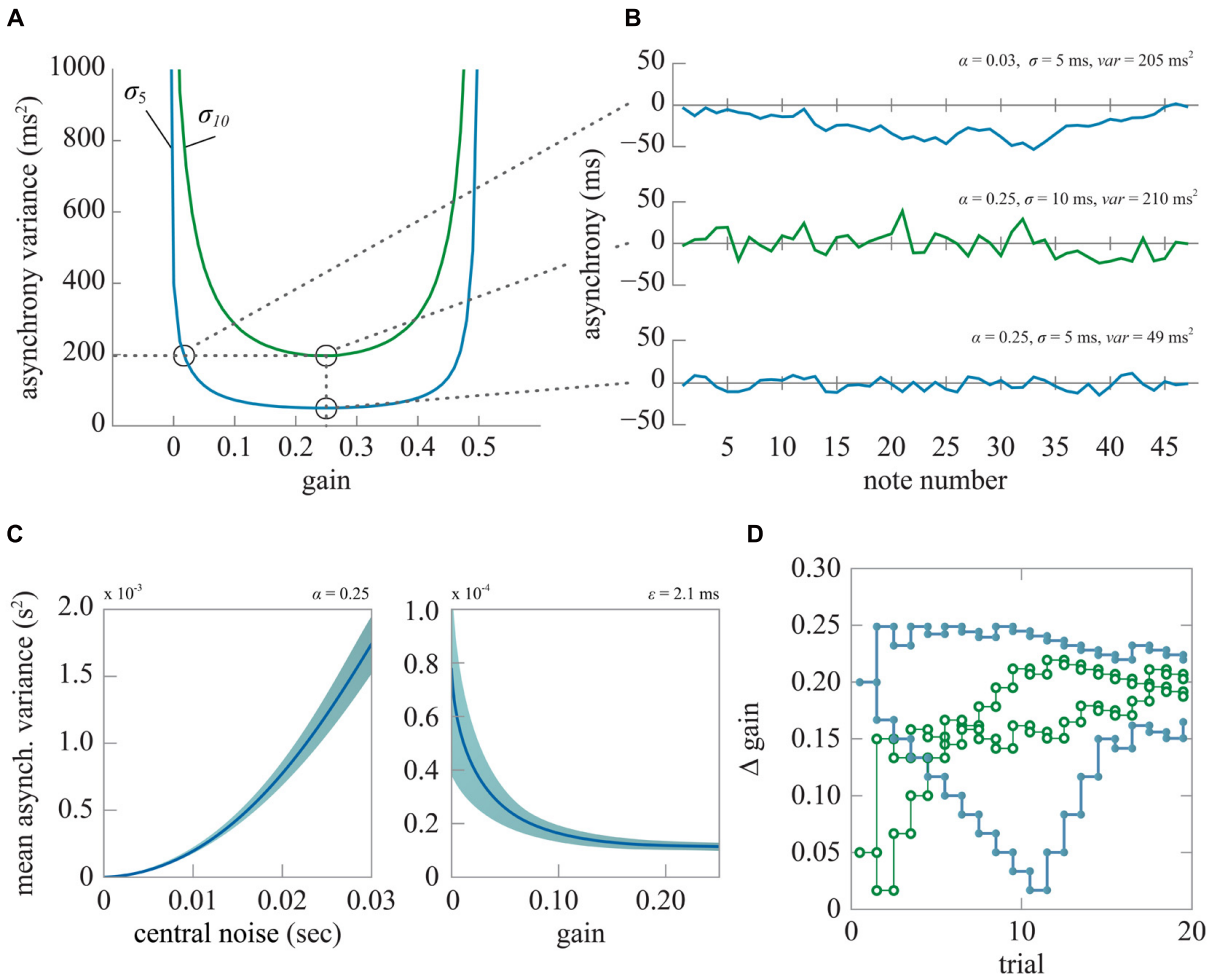
**(A)** Asynchrony variance functions when the timing variability is set to 25 ms^2^ (blue) and 100 ms^2^ (green). The data are averages of 10000 iterations. **(B)** single trial examples of optimal gain with higher (middle) or lower (bottom) timing variability $\sigma_{\epsilon }^{2}$ and less than optimal gain with lower timing variability (top). **(C)** Mean asynchrony variance (±1 SD) of the quartet across 48 notes averaged over 10000 simulations. Left: as a function of timing variability, with gain = 0.25. Right: as a function of gain, with timing variability fixed at 100 ms^2^. **(D)** An example of two sets of staircase functions from two separate blocks for a single participant in Experiment 2. The vertical axis shows the difference in the gain from the non-target stimulus [α = 0.25]. On a given run of trials (green or blue) two staircases with higher (bold line, filled data points) and lower (normal line, empty data points) initial gain differences were presented in alternation on successive trials. In each staircase the gain difference was increased after an incorrect response and reduced after a correct response.

**FIGURE 3:**
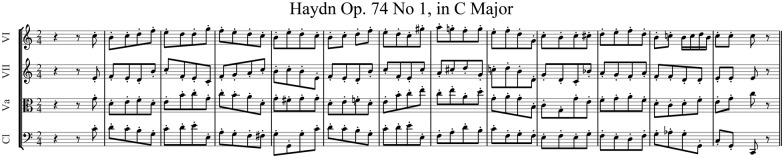
**Excerpt from Haydn Op. 74 No. 1 (fourth movement bars 12–24) used in the listening tests**.

In the first-order linear correction model there are 12 proportional correction gains between all pairs of players. Theoretically, the variance of between-player asynchrony of a quartet is minimized if each player adopts a correction gain of 0.25 (Wing et al., 2014). Smaller or larger gain values are possible, with values in the range 0–0.5 still yielding stable performance, but they lead to greater fluctuations in asynchrony. **Figures 2A,B** shows changes in the asynchrony variance of a virtual quartet performance of a 48-note excerpt of Haydn Op. 74 No. 1 (**Figure 3**) simulated using Eq. (1) with two levels of within-player timing variability and across correction gains, which were set to be equal over all player pairs. It may be seen that the level of the between-player asynchrony variance can be equivalent for a quartet using less than optimal correction gains and a quartet whose members exhibit more within-player timing variability but whose correction gains are optimal (e.g., α = 0.03, $\sigma_{\epsilon }^{2}$ = 25 ms^2^ vs. α = 0.25, $\sigma_{\epsilon }^{2}$ = 100 ms^2^).

In two case studies of professional quartets playing the Haydn excerpt in **Figure 3** we observed gain estimates which, on average, approximated the value of 0.25 (Wing et al., 2014). However, in one quartet the first violin used lower correction gains (0.1) compared to those used by the other players (0.2). This raises the interesting question as to whether a listener could detect the effect of differences in correction gain. Gains smaller (or larger) than 0.25 tend to result in non-zero autocorrelation of the between-player asynchronies at lags of one or higher, whereas the autocorrelation function is critically damped (zero for lags 1 and higher) if gain is equal to 0.25. Such differences in asynchrony autocorrelation might be detectable.

In the research reported in this paper, we asked listeners to identify which of two performances of a musical excerpt appeared less together (less well synchronized). We varied the discrimination task difficulty up and down using a staircase paradigm to determine listeners’ thresholds for detecting differences in between-player asynchrony. In Experiment 1 gains were equal but there was a difference in within-player timing variability, and hence in the between-player asynchrony variability, which, over trials, was adjusted to give 75% correct identification. In Experiment 2 the timing variability was fixed at half the threshold level obtained in Experiment 1 and there was a difference in gains which, over trials, was adjusted to give 75% correct identification. We expected that the resulting difference in asynchrony variance obtained in Experiment 2 would be smaller than that observed in Experiment 1, despite the same accuracy of target identification, on the assumption that the listener is sensitive to the form, and not just the amount, of variance in note asynchrony.

## MATERIALS AND METHODS

Nine participants without specialized musical training (28.6 ± 13.6 years, five males, four right-handed), who provided informed consent according to ethics approved by University of Birmingham ethics committee, took part in the study. In two experiments (Experiments 1, 2), on each trial the participants listened to two samples of a virtual quartet performing the 48 note excerpt shown in **Figure 3** and then reported which sample (the “test” or the “target”) sounded less together (less well synchronized).

We used the first-order linear correction model (Eq. 1) to generate the event times of the virtual quartet at a rate of 162 bpm while the variance of the asynchronies between the players was adjusted by changes in the within-player timing variability ($\sigma_{\epsilon }^{2}$) in Experiment 1 or the correction gain (α) in Experiment 2. In each experiment, the (non-target) test stimulus had zero timing variability ($\sigma_{\epsilon }^{2}$ = 0), and a correction gain of 0.25 set equal across all players. For the test stimulus the only random timing error was in the first note – the resulting error in between-player asynchrony was rapidly compensated over subsequent notes as in Eq. (1). First note onset time was normally distributed with a variance of 25 ms^2^ for all instruments. The target stimulus had non-zero asynchrony variance. This was adjusted on successive trials by an adaptive staircase algorithm using accelerated stochastic approximation (Kesten, 1958) to adjust step size and converge on the level of timing variability (Experiment 1) or gain (Experiment 2) that gave 75% correct target identification (**Figure 2D**). Feedback was given at the end of each trial as to whether the target stimulus had been correctly identified.

**FIGURE 4:**
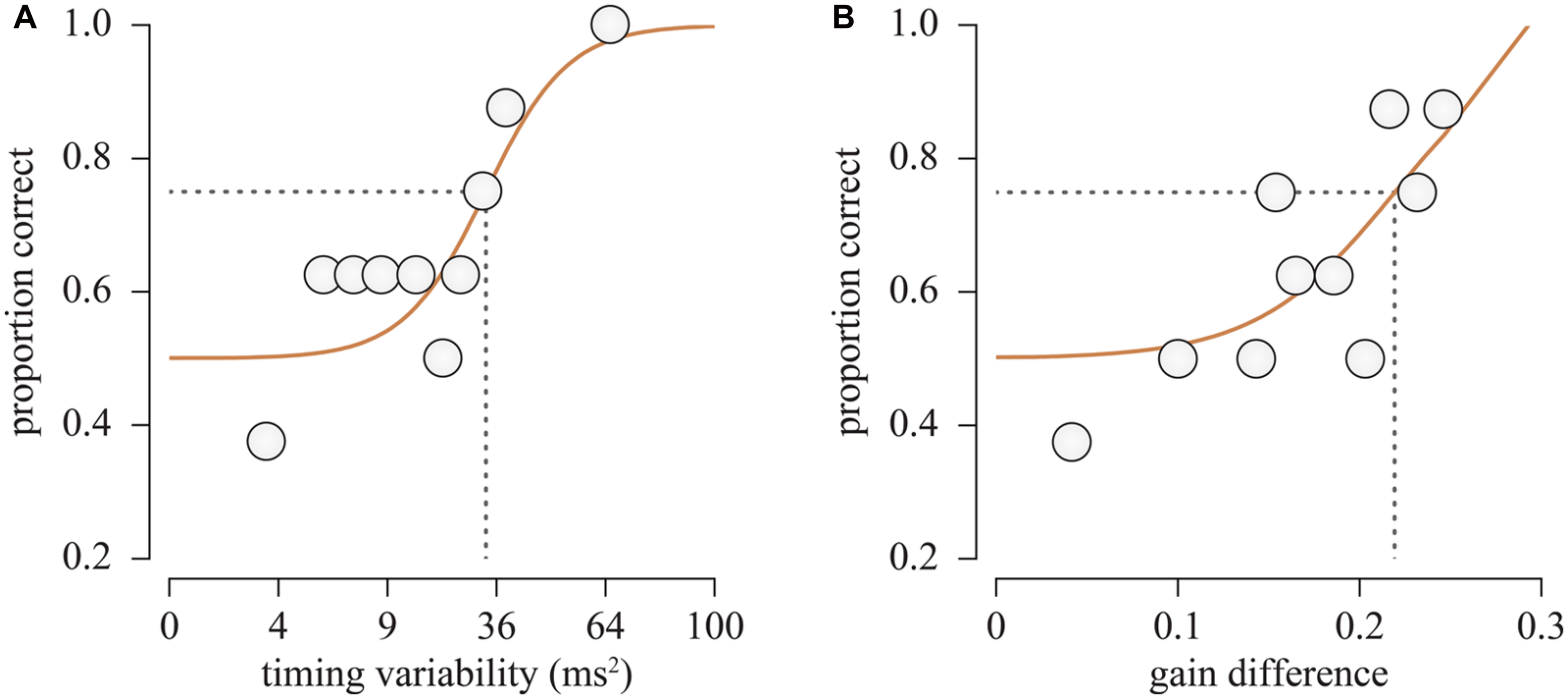
**Binned proportion of correct responses as a function of (left) the timing variability (Experiment 1) and (right) gain difference in non-target and target quartets (Experiment 2) calculated for a single participant**.

Due to the random nature of the stimulus generation process, asynchrony variance can vary between stimuli generated using identical within-player timing variability (Experiment 1) or gain (Experiment 2) as illustrated in **Figure 2C**. To ensure that the between-player asynchrony variance of the stochastically generated stimulus was close to the theoretical value for the timing variability or gain value specified by the staircase, 10000 sets of time-series were generated for each set of simulation parameters before the experiment, and the mean asynchrony variance was determined at each level. The required simulation parameters for given asynchrony variances were then calculated using linear interpolation between the simulated mean asynchrony variances. If the resulting generated stimulus asynchrony variance (calculated per note, and averaged over all 48 notes) was outside a tolerance region of the theoretical variance plus or minus 10^-7^ s^2^, then new stimuli were generated until one resulted with asynchrony variance within the tolerance region. In Experiment 1 the asynchrony variance threshold at which the listener correctly identified the target on 75% of trials corresponding to [$\sigma_{\epsilon }^{2}$ = ThCENTRAL, α = 0.25] was measured by fitting the logistic function to the psychometric data consisting of binned fractional correct responses (**Figure 4**). In Experiment 2, the timing variability was fixed at half the level, i.e., [$\sigma_{\epsilon }^{2}$ = ThCENTRAL/2] corresponding to the threshold determined in Experiment 1, and the gain was controlled by the staircase algorithm to converge on 75% correct target identification.

The simulated time series data were combined with note pitch and duration information to create a midi file using the Midi Toolbox in MATLAB (Eerola and Toiviainen, 2004). Velocity was set at 80 and there was no variation in loudness across notes or between instruments. The duration of the eighth notes was 50 ms and staccato was expressed by halving the note duration. The generated stimuli were presented using a standard media player (winamp/windows media player) through a pair of headphones (Audio Technica, ATH-M30/ Sennheiser) at a comfortable volume in a quiet room. Participants performed 80 trials divided into two blocks per experiment and, altogether, the experiment took approximately 80 min to complete.

## RESULTS

The average tempo for the stimuli generated in Experiments 1 and 2 was 162.0 ± 3.3 bpm. For each participant, responses were divided into 10 bins by staircase levels, and the mean fraction of correct response was calculated per bin. The proportion of correct responses was then fitted with a logistic function and the 75% correct response level was determined as the detection threshold of between-player asynchrony variance (see **Figure 4**; **Table 1**). In Experiment 1, the mean asynchrony variance of the target sample at threshold was 64.3 ms^2^ (SD = 52.0 ms^2^). The threshold was reduced to an average of 18.2 ms^2^ (SD = 12.8 ms^2^) when the time series of the asynchronies between pairs of players were altered by the introduction of the varied correction gains in Experiment 2. A paired-sample *t*-test (after taking natural logarithms to normalize the distributions) confirmed that the threshold in Experiment 2 was lower than the threshold in Experiment 1, *t*(8) = 7.978, *p* < 0.0005. The log detection threshold of the asynchrony variance for each participant in Experiment 1 was not reliably correlated (*r* = 0.08, *p* = 0.823) with the sensitivity to the gain difference as defined by the threshold reduction rate in Experiment 2 [1-var(Experiment 2)/var(Experiment 1)].

**Table 1:** Threshold expressed in terms of variance of asynchrony (ms^2^).

Participant	Experiment 1	Experiment 2
1	62.3	17.0
2	193.6	48.4
3	66.9	11.5
4	82.9	21.1
5	32.3	8.9
6	32.8	8.9
7	34.9	12.9
8	42.8	8.9
9	29.9	26.3

Mean	64.3 (52.0)	18.2 (12.8)

Experiment 2 differed from Experiment 1 in that the correction gain was varied, which, in turn, affected the nature of variation in the asynchronies between players as revealed by the lag one autocorrelation. The largest of the lag one autocorrelations for the six pairs of between-player asynchronies was selected on each trial. The autocorrelation was reliably larger in Experiment 2 (0.84, SD = 0.05) than Experiment 1 (0.39, SD = 0.024 ), [*t*(8) = –20.848, *p* < 0.0005].

## DISCUSSION

In a typical small group ensemble, the different instruments exhibit small variations in timing and this results in variable aysnchrony between the note onsets of the various player pairs. Rasch (1979, 1998) documented SDs of between-player asynchrony in a string and a woodwind trio in the range 24–73 ms while Wing et al. (2014) reported values within this range in two string quartets. Such variability in between-player asynchrony is a challenge for the maintenance of ensemble and it was proposed that string quartets accordingly employ first-order linear correction of asynchrony between all pairs of players (Wing et al., 2014). In this quartet timing model, a correction value or gain of 0.25 is optimal in the sense of minimizing the variability of asynchrony. However, gains can range over 0–0.5 and still produce stable performance such that a synchronization error between two players is quickly reduced over the next few notes. If the gain lies between 0 and 0.25, the asynchrony correction is overdamped (the reducing error has the same sign over successive notes) and if between 0.25 and 0.5, the correction is underdamped (the reducing error alternates in sign). When the gain is 0.25 the correction is critically damped in that it is fully eliminated on the next note. The question this poses is whether differences in correction gain are discriminable to the listener through the pattern of asynchrony errors?

In this paper we present a method for determining the threshold at which listeners can discriminate between two virtual quartets, simulated using the first-order linear correction model. Using this method, Experiment 1 reveals that when the correction gains are optimal, the average threshold for discriminating between two musical excerpts differing only in between-player asynchrony variability is appreciably less (by a factor of 5 in terms of SD) than the published estimates of ensemble asynchrony variability referred to above. This suggests that listeners would be able to distinguish between typical quartets on the basis of differences in their asynchrony variability. Moreover, in Experiment 2, when the two musical excerpts differed in correction gain, they were discriminated at a point where the difference in asynchrony variability was at a level well below (by a factor of 2 in terms of SD) the threshold estimated in Experiment 1. This suggests that listeners are sensitive to the form of correction, probably because of the structure present in the variability of the asynchronies. For example, consider when the gain approaches zero, when discrimination against optimal gain becomes more reliable. The asynchronies exhibit persistent positive or negative departures from average (reliably larger lag one autocorrelation in Experiment 2 compared to Experiment 1), producing between player shifts in phase, and this may have been the cue used by participants.

In Experiment 2 we examined correction gains in the range 0 to 0.25. Gains in this range produce damped convergence on zero asynchrony error. It would be interesting to know whether correction gains between 0.25 and 0.5, which result in alternation in the sign of the asynchrony as it reduces to zero with successive notes, would be more noticeable and lead to better discrimination of the target stimulus. It might be supposed that correction gains set by the individual player are subject to error. If deviations from the optimal value of 0.25 are less perceptible for the listener than when gains are greater than 0.25, players might adopt the strategy of setting their target gain less than 0.25. However, there are other possible reasons for setting the gain less than 0.25. For example, if there is appreciable variability in neuromuscular performance delays, the optimal value decreases with increasing motor variability (Burge et al., 2008).

Our method of obtaining discriminability estimates involved a very low level of variability (with optimal correction gain) in the test stimulus. This might be considered unrealistic for musical contexts where the discrimination task would typically involve identifying the more variable of two quartets, where both have appreciable asynchrony variability. The ability to discriminate target from test is likely to decline with increase in baseline variability by analogy with the increase in threshold for duration discrimination with duration (Getty, 1975). In future research it will be interesting to examine a musically more realistic situation in which the baseline variability of the test stimulus corresponds to the variability of a professional quartet and to determine the just noticeable difference with increased variability in the target quartet. Further methodological adjustments for greater musical realism could involve the addition of timing complexities such as rhythm, non-homophony (which would require a beat- or pulse-based rather than note-based unit of analysis) and variation in dynamics. If the additional stimulus dimensions are uncorrelated with asynchrony variability, judgments about asynchrony variance might become harder due to attention demands, parelleling, for instance, the effects of judging intensity on concurrent judgments of duration (Casini and Macar, 1997).

In summary we have presented new listening tests for the ability to discriminate variability of note onset asynchrony in string quartet performance. The results of the first experiment show that people discriminate differences in asynchrony variability that are appreciably less than the variability typical of professional quartets. The second experiment shows that discrimination is further improved if the performances differ in the correction gain employed to counteract timing variability and maintain ensemble.

## Conflict of Interest Statement

The authors declare that the research was conducted in the absence of any commercial or financial relationships that could be construed as a potential conflict of interest.
